# FOXA2 alleviates CCl_4_-induced liver fibrosis by protecting hepatocytes in mice

**DOI:** 10.1038/s41598-017-15831-6

**Published:** 2017-11-14

**Authors:** Wei Wang, Li-Jia Yao, Weifeng Shen, Kai Ding, Pei-Mei Shi, Fei Chen, Jin He, Jin Ding, Xin Zhang, Wei-Fen Xie

**Affiliations:** 1 Department of Gastroenterology, Changzheng Hospital, Second Military Medical University, 415 Fengyang Road, Shanghai, 200003 China; 2 Department of Special Treatment, Eastern Hepatobiliary Surgery Hospital, Second Military Medical University, 225 Changhai Road, Shanghai, 200438 China; 3 International Cooperation Laboratory on Signal Transduction of Eastern Hepatobiliary Surgery Institute, Second Military Medical University, Shanghai, China; 4 Present Address: Department of Gastroenterology, Lanzhou General Hospital of Lanzhou Military Command, Lanzhou, China; 5 Present Address: Department of Gastroenterology, Fuzhou General Hospital, Fuzhou, 350025 China

## Abstract

The liver-enriched transcription factor Forkhead Box A2 (FOXA2) has been reported to be involved in bile acid homeostasis and bile duct development. However, the role of FOXA2 in liver fibrogenesis remains undefined. In this study, we found that the abundance of FOXA2 was significantly lower in fibrotic livers of patients and mice treated with CCl_4_ than in controls. Interestingly, the expression level of FOXA2 decreased in hepatocytes, whereas FOXA2 was elevated in hepatic stellate cells (HSCs) of mouse fibrotic livers. Hepatocyte-specific ablation of FOXA2 in adult mice exacerbated liver fibrosis induced by CCl_4_. Either lentivirus LV-CMV-FOXA2 mediated FOXA2 overexpression in the liver or adeno-associated virus AAV8-TBG-FOXA2-mediated hepatocyte-specific upregulation of FOXA2 alleviated hepatic fibrosis. Overexpression of FOXA2 in HSCs did not obviously affect hepatic fibrogenesis. Additionally, FOXA2 knockout in hepatocytes resulted in aberrant transcription of metabolic genes. Furthermore, hepatocyte-specific knockout of FOXA2 enhanced endoplasmic reticulum stress (ER stress) and the apoptosis of hepatocytes, whereas FOXA2 overexpression in hepatocytes suppressed ER stress and hepatocyte apoptosis in mouse fibrotic livers. In conclusion, our findings suggested that FOXA2-mediated hepatocyte protection has a therapeutic role in hepatic fibrosis, and thus may be a new, promising anti-fibrotic option for treating chronic liver diseases.

## Introduction

Various chronic liver injuries induced by persistent infections, alcohol abuse, chemical insults, and metabolic or autoimmune reactions can give rise to fibrosis, cirrhosis, liver failure and even tumour formation^1–3^. Hepatic fibrosis is an early stage of cirrhosis that is a result of pathological deposition of extracellular matrix (ECM) in the liver. It has been well documented that the activation of hepatic stellate cells (HSCs) is the key event in hepatic fibrogenesis^4^. Stimulation of chronic injury may lead to perpetuation and acceleration of HSC activation with increased ECM synthesis and impaired ECM degradation (fibrolysis), thus resulting in the disruption of the normal liver architecture and ultimately in cirrhosis^3^. Therefore, activated HSCs have become an attractive target for antifibrotic therapy in the past few decades. However, recent studies have indicated that HSCs also play a critical role in the process of liver development and regeneration^5^. A variety of mitogenic factors produced by HSCs promote liver regeneration by affecting hepatocytes, progenitor cells or bone marrow-derived mesenchymal stem cells (MSCs)^6,7^. In addition, HSCs play a potential role in liver regeneration through transdifferentiation into liver progenitor cells^8^. Thus, anti-fibrosis therapy targeted to HSCs may affect liver regeneration.

Mammalian hepatocyte nuclear factors (HNFs), including HNF1, HNF3, HNF4, HNF6 and CCAAT/enhancer-binding proteins, form a transcriptional network controlling hepatocyte differentiation and function during embryonic development and liver homeostasis in adults^9^. Our previous studies have demonstrated that forced expression of either HNF1α or HNF4α alleviates hepatic fibrosis by protecting hepatocytes against damage or inhibiting epithelial-mesenchymal transition (EMT) in hepatocytes^10,11^. Remarkably, reprogramming the transcription factor network in hepatocytes by HNF4α reverses dysfunctional hepatocytes and hepatic failure^12^. Together, these data suggest that HNFs may be potential therapeutic targets for treating liver fibrosis.

Forkhead box A2 (FOXA2), also known as HNF3β, is one of the transcriptional regulators of the HNF3 family^13^. FOXA2 cooperates with FOXA1 (also known as HNF3α) to establish competence in the foregut endoderm and is required for the initiation of liver development^14^. Both FOXA1 and FOXA2 are positive regulators of bile duct development in mice^15^. In the adult liver, FOXA2 is critical for glucose and lipid homeostasis^16,17^. The quantity of FOXA2 appears to steadily decline in liver injury with various aetiologies^18^. Deletion of FOXA2 in the liver at late gestation leads to decreased transcription of genes encoding bile acid transporters and conjugation enzymes, thus disturbing bile acid homeostasis, endoplasmic reticulum (ER) stress and liver injury^19^. In addition, one recent study has demonstrated that FOXA2 mediates the therapeutic effects of biliary-committed progenitor cells during cholestatic liver injury^20^. More importantly, FOXA1 and FOXA2 have been found to be essential for sexual dimorphic hepatocellular carcinoma (HCC) in mice^21^. Our recent study has revealed an inhibitory effect of FOXA2 in the metastasis of hepatocellular carcinoma^22^. However, there is no direct evidence showing a relationship between FOXA2 and liver fibrosis.

In this study, we generated mutant mice in which FOXA2 was specifically deleted in hepatocytes and demonstrated that FOXA2 knockout in hepatocytes exacerbated liver fibrosis induced by CCl_4_. Additionally, by using three distinct viruses to deliver the expression of FOXA2 into the liver, we found that FOXA2 attenuated liver fibrosis by protecting hepatocytes from endoplasmic reticulum stress (ER stress) and apoptosis.

## Results

### FOXA2 expression is downregulated after hepatic fibrogenesis

**Figure 1:**
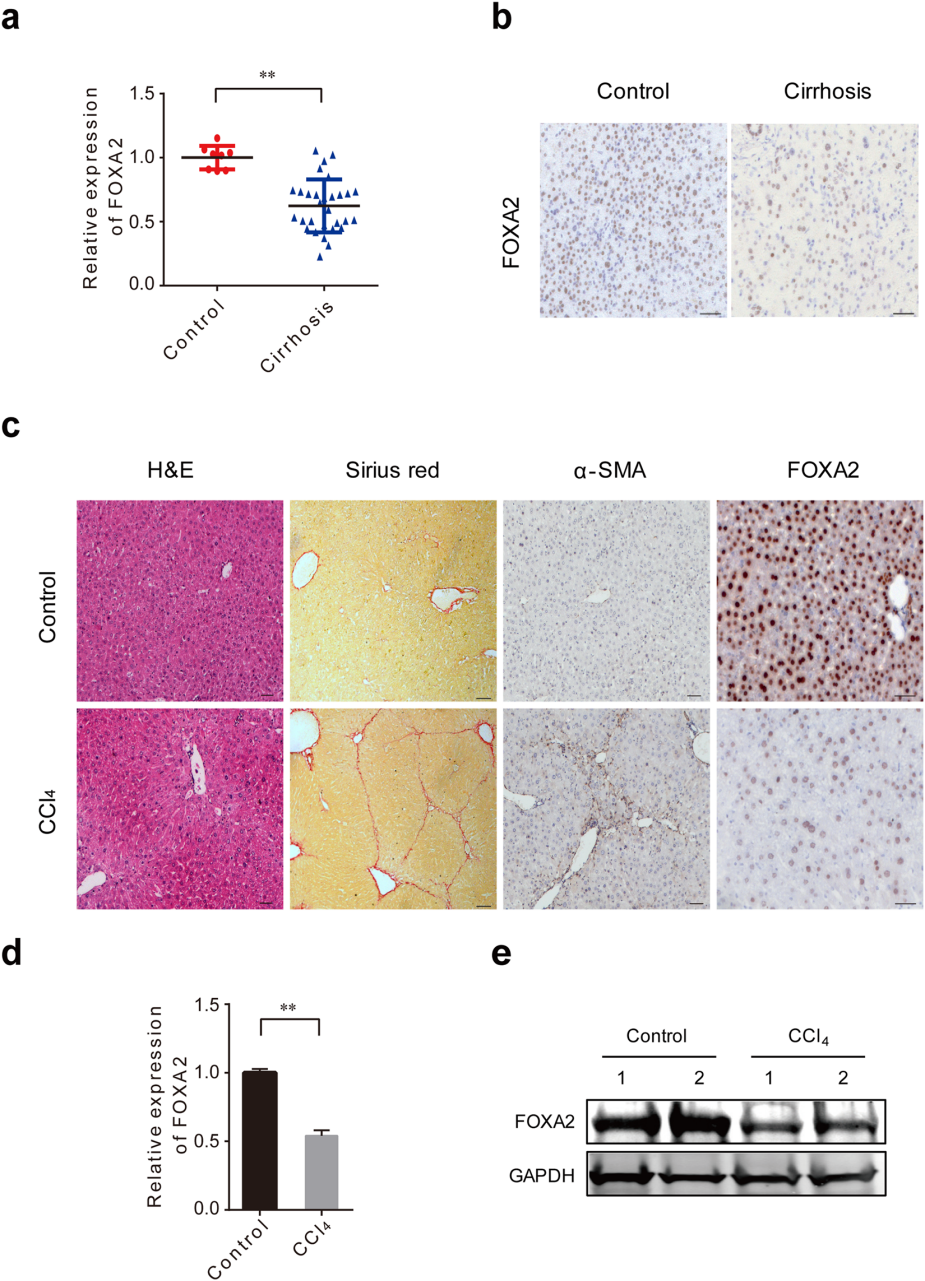
FOXA2 expression is lower in fibrotic livers. (**a**) A scatter dot plot showing FOXA2 expression in 8 human controls and 30 fibrotic or cirrhotic specimens, as evaluated by PCR analysis. The black horizontal line in the middle indicates the median value. Data are normalized to β-actin, and the *P* value was calculated by Wilcoxon’s matched pair test (*P* = 0.0079). **P < 0.01. (**b**) Immunohistochemistry was carried out to assess FOXA2 expression in the livers from patients. Scale bars, 50 μm. (**c**) The expression of FOXA2 and the degree of fibrosis in the liver tissues of the CCl_4_-treated mice are shown through immunohistochemistry, Haematoxylin and eosin (H&E) and Sirius red staining. Scale bars, 50 μm. (**d**) The mRNA levels of FOXA2 in the livers were detected by real-time PCR. **P < 0.01. (n = 6 in each group) (**e**) The protein levels of FOXA2 from two individual mouse livers of 5-week CCl_4_-treated mice and normal controls were detected by western blotting.

To explore the role of FOXA2 in hepatic fibrosis, we first examined FOXA2 expression levels in the livers of 8 human controls and 30 patients with liver fibrosis or cirrhosis by using RT-PCR. The results revealed that FOXA2 was significantly downregulated in fibrotic livers compared with controls (Fig. 1a). Immunohistochemistry indicated only faint staining of FOXA2 in the nuclei of cells in human fibrotic livers, whereas FOXA2 was clearly present in the nuclei of controls (Fig. 1b). We then evaluated the expression of FOXA2 in mouse fibrotic livers. Male C57/B6 mice were injected intraperitoneally with CCl_4_ biweekly for 5 weeks to establish a hepatic fibrosis mouse model. Along with these fibrotic changes, staining with H&E and Sirius red confirmed collagen accumulation, a characteristic of liver fibrosis (Fig. 1c). As expected, FOXA2 also significantly declined in mouse livers during the progression of hepatic fibrosis (Fig. 1c,d and e), a result consistent with alterations in humans. Together, these findings indicated that progressive liver fibrosis is associated with decreased FOXA2 expression.

### FOXA2 expression is downregulated in hepatocytes and upregulated in activated HSCs during liver fibrogenesis

**Figure 2:**
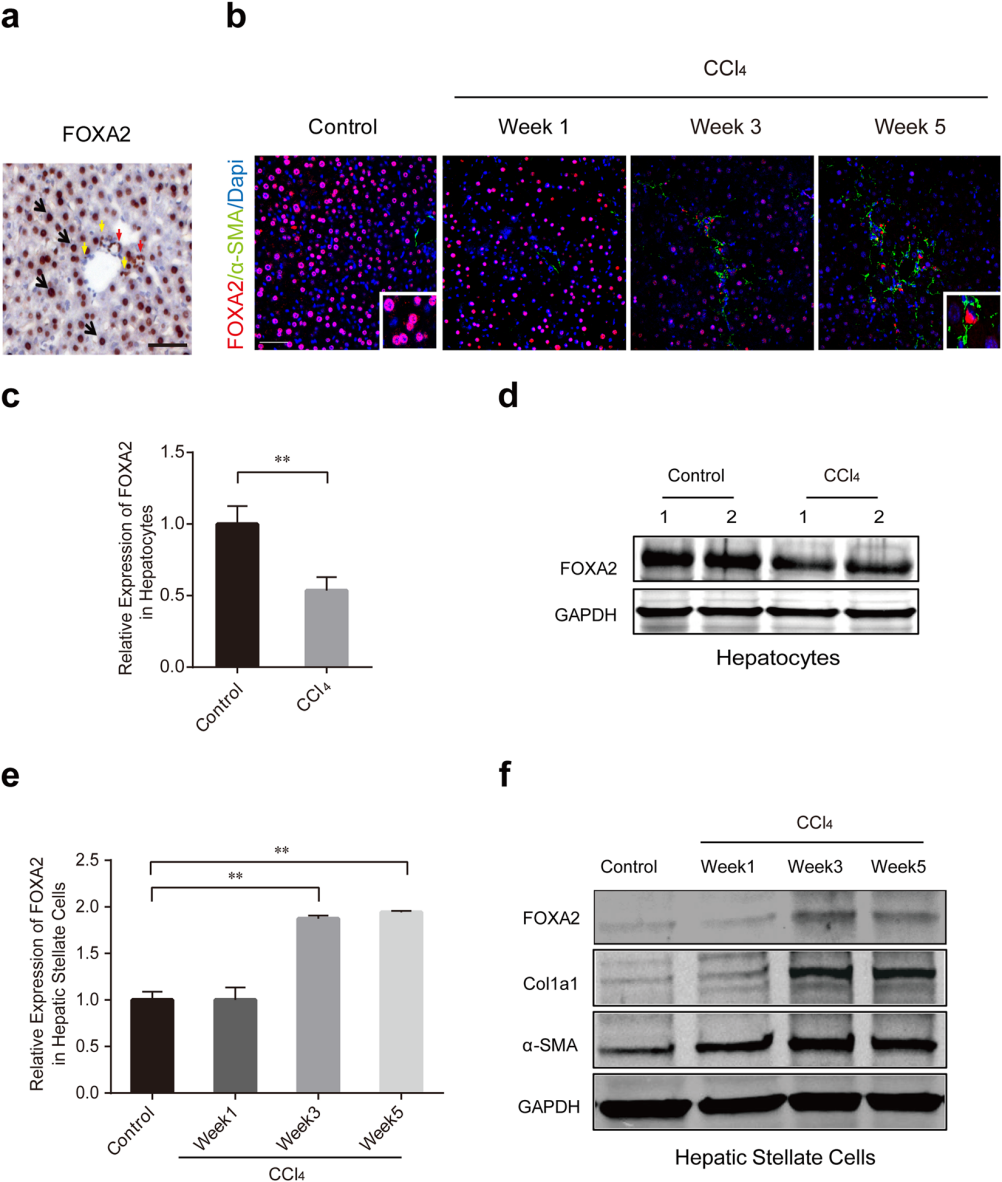
FOXA2 is downregulated in hepatocytes but upregulated in HSCs in fibrotic livers. (**a**) Immunohistochemical staining of FOXA2 in normal mouse livers. FOXA2 was detected in the nuclei of hepatocytes (black arrow), cholangiocytes (red arrow) and other non-parenchymal cells (yellow arrow). Scale bar, 50 μm. (**b**) Immunofluorescence staining showing the levels of FOXA2 (red) and α-SMA (green) in the hepatic tissues from control and CCl_4_-treated mice. The cell nuclei (blue) were counterstained with DAPI. Scale bars, 50 μm. (**c**,**d**) Real-time PCR and western blot analysis of FOXA2 expression in the primary hepatocytes derived from normal control individuals and fibrosis livers treated with CCl_4_ for 5 weeks. (**e**) The mRNA level of FOXA2 and (**f**) the protein level of FOXA2, α-SMA and Col1a1 in the HSCs isolated from control and fibrotic liver tissues after CCl_4_ injection were detected (n ≥ 3 in each group). **P < 0.01.

Immunohistochemistry revealed that both hepatocytes and non-parenchymal cells expressed FOXA2 in normal mouse livers (Fig. 2a). Interestingly, immunofluorescence staining revealed that FOXA2 expression was decreased in hepatocytes and increased in activated HSCs during liver fibrogenesis (Fig. 2b). Furthermore, the levels of FOXA2 mRNA and protein in primary hepatocytes isolated from fibrotic mice were also markedly decreased (Fig. 2c and d). In contrast, compared with the quiescent HSCs from normal livers, the activated HSCs isolated from fibrotic livers expressed a dramatically higher level of FOXA2 (Fig. 2e and f). Collectively, these data indicated that FOXA2 may play distinct biological roles in hepatocytes and HSCs during hepatic fibrosis.

### Deletion of FOXA2 in hepatocytes aggravates hepatic fibrosis

**Figure 3:**
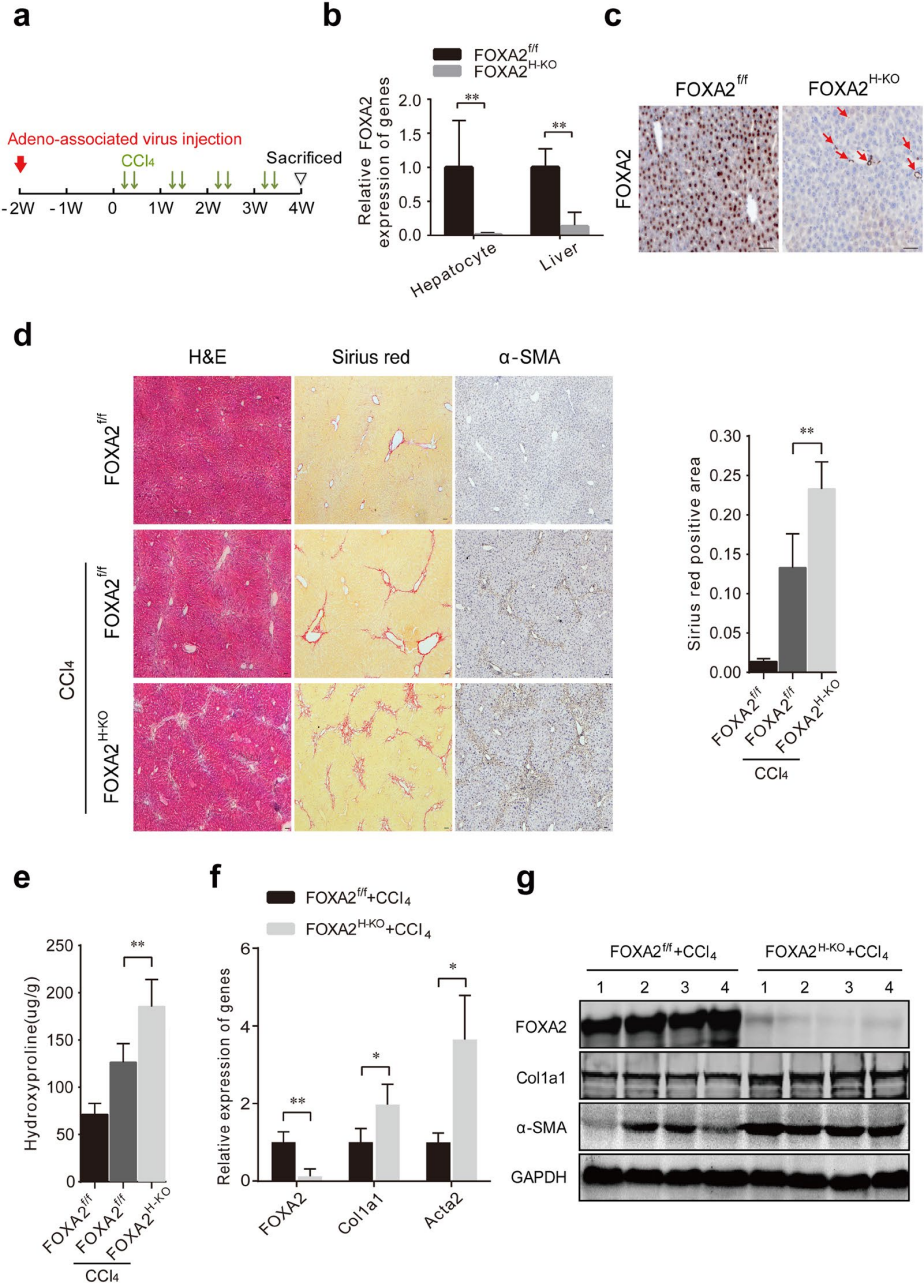
Hepatocyte-specific ablation of FOXA2 promotes liver fibrosis in mice. (**a**) Schematic representation of induction of hepatic fibrosis. Adeno-associated virus AAV8-TBG-Control or AAV8-TBG-Cre was injected via the tail vein of FOXA2^flox/flox^ (FOXA2^f/f^) mice 2 weeks before CCl_4_ administration. Hepatic fibrosis was induced by injection of CCl_4_ two times per week for 4 weeks. The mice were sacrificed on the third day after the last injection of CCl_4_. (**b**) The FOXA2 mRNA in hepatocytes and livers of adult FOXA2^f/f^ and FOXA2^H-KO^ mice was analysed by real-time PCR (n = 4). (**c**) The expression of FOXA2 was determined by using immunohistochemistry. FOXA2 was almost absent in the hepatocytes of FOXA2^H-KO^ mice. Residual FOXA2 staining remained in the nuclei of non-parenchymal cells (red arrow). Scale bars, 50 μm. (**d**) Representative sections from livers stained for α-SMA using immunohistochemistry. Liver morphology in wild type (WT), control and mutant fibrotic mice was analysed with H&E and Sirius red staining. Scale bars, 50 μm. Right panel: Semi-quantitative analysis of Sirius red staining in the fibrotic livers of each group. (**e**) The amount of hydroxyproline in the fibrotic livers was significantly increased in mice with FOXA2 gene excision. (**f**,**g**) The mRNA levels (**f**) and the protein levels (**g**) of FOXA2, Col1a1 and Acta2 in the livers were assessed by real-time PCR and western blotting (n = 6 mice in each group). *P < 0.05, **P < 0.01, ***P < 0.001.

To examine the function of FOXA2 in hepatocytes after liver fibrogenesis, hepatocyte-specific FOXA2-deficient (FOXA2^H-KO^) mice were created by using Cre-loxP-based recombination. Replication-incompetent AAV8-TBG-Cre virus for Cre expression under the control of hepatocyte-specific thyroid-binding globulin (TBG) promoter was used for hepatocyte-specific expression of Cre recombinase^23^. Adult FOXA2^f/f^ mice were injected with AAV8-TBG-Cre to knock out FOXA2 specifically in hepatocytes (Fig. 3a). Real-time PCR analysis revealed that FOXA2 expression was eliminated in hepatocytes but retained in non-parenchymal cells (duct cells, HSCs and endothelial cells, etc.) in the livers of FOXA2^H-KO^ mice (Fig. 3b); this finding was further confirmed by immunohistochemistry (Fig. 3c). The liver fibrosis was induced by a 4-week treatment of CCl_4_ (Fig. 3a). The liver weight is significantly decreased in the FOXA2^H-KO^ fibrotic mice compared with the FOXA2^f/f^ fibrotic mice (Supplementary Fig. 1a). As shown in Fig. 3d, the fibrotic livers of FOXA2^H-KO^ mice had more ECM deposition than the livers treated with control virus. FOXA2^H-KO^ increased the ECM area by 75.32% in the fibrotic model compared with that in controls (*P* < 0.01) (Fig. 3d). Meanwhile, the FOXA2^H-KO^ group had much higher hydroxyproline content compared with the control group (185.52 ± 24.87 μg/mg vs 126.63 ± 17.92 μg/mg, *P* < 0.01) (Fig. 3e). In addition, the α-SMA level was increased by FOXA2 deletion, suggesting that the activation of HSCs was enhanced (Fig. 3d). Similarly, the mRNA levels of the fibrotic markers Acta2 (encoding α-SMA) and Col1a1 (encoding a1 (I) collagen) were notably upregulated in the fibrotic livers of FOXA2^H-KO^ mice (Fig. 3f); this finding coincided with the high protein expression of the fibrotic genes shown in the western blot (Fig. 3g). Together, these data indicated that hepatocyte-specific excision of FOXA2 promotes liver fibrosis in mice.

### FOXA2 overexpression in the liver mitigates hepatic fibrosis

**Figure 4:**
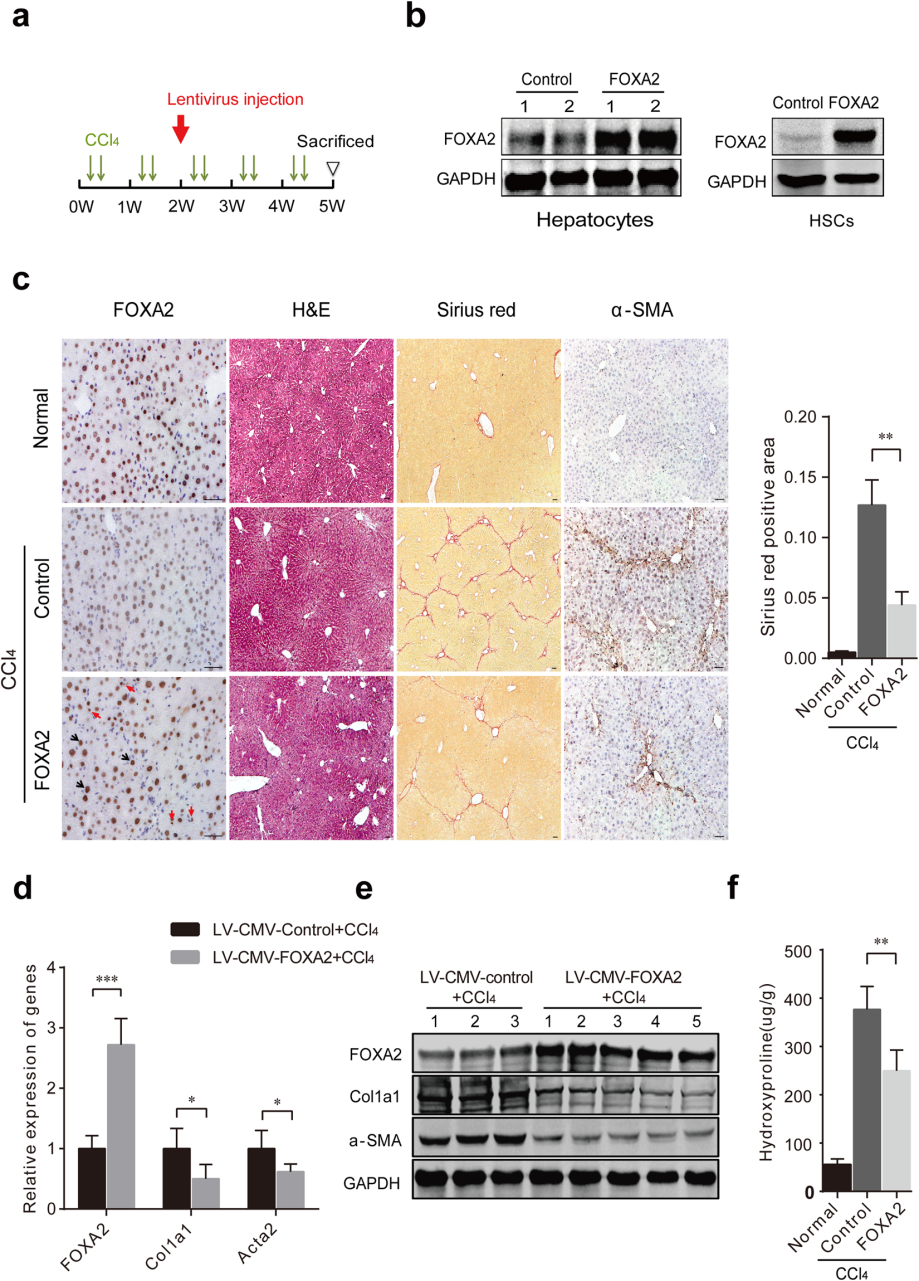
Upregulation of FOXA2 in the liver ameliorates fibrogenesis. (**a**) A single dose of a lentivirus carrying the FOXA2 gene or a single dose of control virus was injected via the tail vein of C57Bl/6 J mice 2 weeks after the first CCl_4_ injection. The fibrotic livers from the mice were analysed 5 weeks after CCl_4_ treatment. (**b**) Western blot for FOXA2 protein expression in primary hepatocytes and HSCs isolated from mice treated with control virus or LV-CMV-FOXA2. (**c**) The expression levels of FOXA2 and α-SMA in the fibrotic livers were assessed by immunohistochemistry. Enhanced FOXA2 expression was located in hepatocytes (black arrow) and non-parenchymal cells (red arrow) in the liver injected with LV-CMV-FOXA2. H&E and Sirius red staining were used to examine the pathological alterations and collagen deposition. Semi-quantitative analysis of Sirius red staining in the fibrotic livers after FOXA2 gene delivery was compared with that in controls (right). Scale bars, 50 μm. (**d**,**e**) The mRNA levels (**d**) and the protein levels (**e**) of FOXA2, fibrogenic markers (Acta2 and Col1a1) in the fibrotic livers were detected by real-time PCR and western blotting, respectively. (**f**) The hydroxyproline content in mouse livers from each group (n = 6 mice in each group). *P < 0.05, **P < 0.01, ***P < 0.001.

Because deletion of FOXA2 in hepatocytes exacerbates the development of hepatic fibrosis, we wondered how FOXA2 upregulation might affect liver fibrosis. Lentivirus for FOXA2 expression under the control of the CMV promoter, LV-CMV-FOXA2, was used to upregulate the expression of FOXA2 in the liver (Fig. 4a). As expected, both hepatocytes and HSCs exhibited significantly higher FOXA2 expression after FOXA2 delivery (Fig. 4b). In addition, a single dose of LV-CMV-FOXA2 injection dramatically enhanced FOXA2 expression in the fibrotic livers and increased the liver weight of the mice with 5-week CCl_4_ treatment (Fig. 4c–e and Supplementary Fig. 1b). Intriguingly, LV-CMV-FOXA2 administration inhibited the development of hepatic fibrosis, as confirmed by H&E and Sirius red staining (Fig. 4c). Quantification indicated that the Sirius red-positive area was significantly lower (by 65.32%) in the fibrotic livers of the mice with LV-CMV-FOXA2 delivery than that in the livers of the control virus group (*P* < 0.01) (Fig. 4c). Furthermore, a quantitative analysis indicated that, compared with hydroxyproline in the livers of the controls (376.81 ± 43.37 μg/mg), hydroxyproline in livers receiving LV-CMV-FOXA2 (249.93 ± 38.40 μg/mg,* P *< 0.01) was significantly lower (Fig. 4f). The accumulation of Acta2 and Col1a1 in fibrotic livers was also suppressed by FOXA2 overexpression (Fig. 4c–e).

### Hepatocyte-specific overexpression of FOXA2 alleviates liver fibrosis

**Figure 5:**
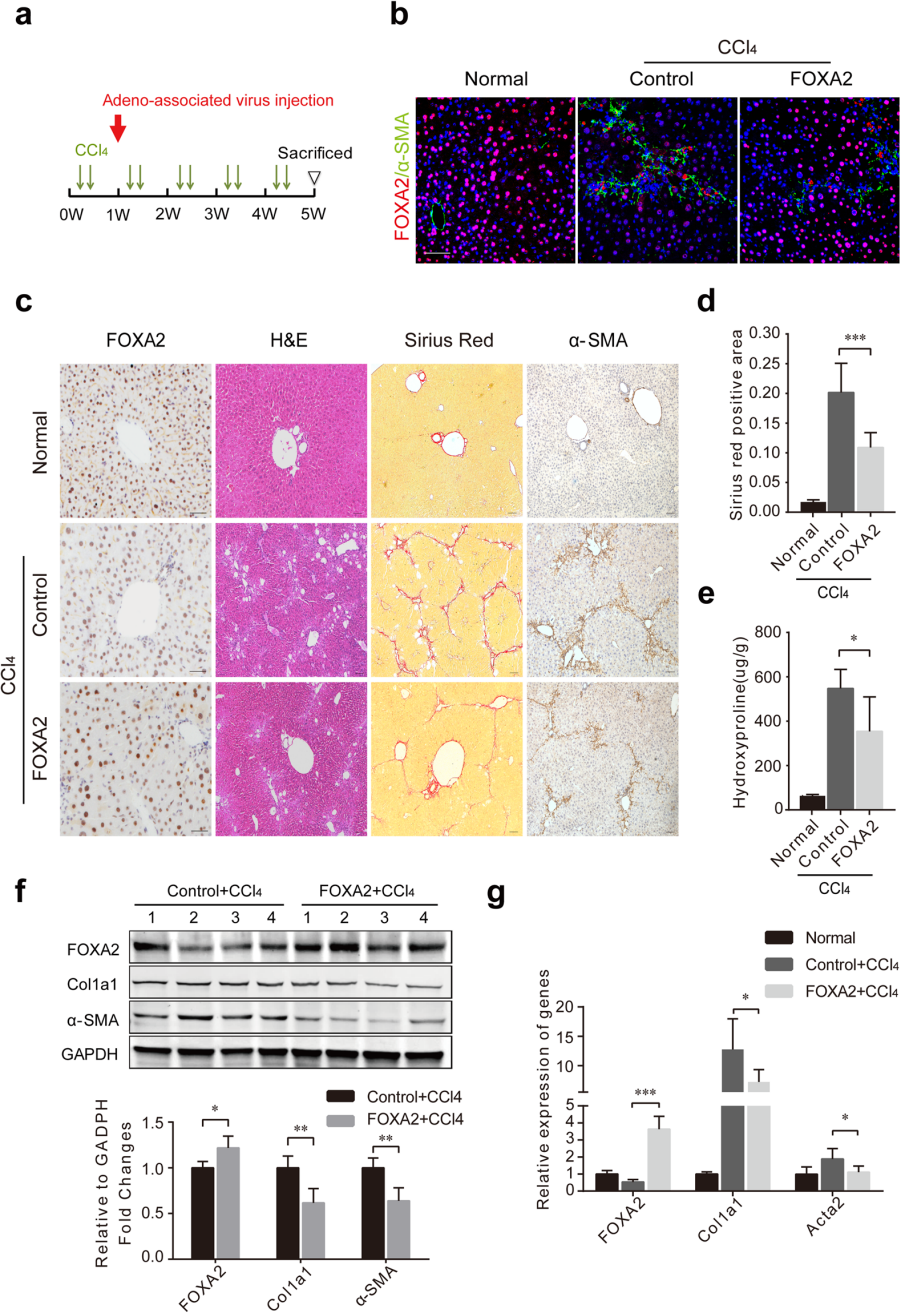
Hepatocyte-specific overexpression of FOXA2 alleviates liver fibrosis. (**a**) The hepatocyte-specific FOXA2 overexpression model was generated by injecting virus AAV8-TBG-FOXA2 via the tail vein in C57Bl/6 J mice 1 week after the first CCl_4_ injection. Mice were sacrificed 5 weeks after CCl_4_ administration. (**b**) Immunofluorescence staining showed the expression of FOXA2 and α-SMA. More staining for FOXA2 was located in hepatocytes after FOXA2 gene delivery. Scale bars, 50 μm. (**c**) Immunohistochemistry staining for FOXA2 and α-SMA. H&E and Sirius red staining were performed to evaluate the pathological alterations and ECM deposition. Scale bars, 50 μm. (**d**) Semi-quantitative analysis of Sirius red staining of the livers from each group mice. (**e**) The amount of hydroxyproline in mouse livers. (**f**,**g**) The protein levels (**f**) and the mRNA levels (**g**) of FOXA2 and fibrogenic genes (Col1a1 and Acta2) in mouse livers (n = 8–10 mice in each group). *P < 0.05, **P < 0.01, ***P < 0.001.

To further explore the effects of hepatocyte expression of FOXA2 on fibrosis, a single dose of AAV8-TBG-FOXA2 or the control virus AAV8-TBG was injected into the mice 1 week after the first CCl_4_ injection. The mice were sacrificed after a 5-week CCl_4_ treatment (Fig. 5a). Immunofluorescence and immunohistochemistry revealed more pronounced FOXA2 staining in hepatocytes in the mice treated with AAV8-TBG-FOXA2 (Fig. 5b and c). In addition, hepatocyte-specific upregulation of FOXA2 in the livers resulted in lower ECM deposition (by 45.94%) than that in the livers of control mice (Fig. 5c and d). Moreover, FOXA2 delivery to hepatocytes also resulted in lower hydroxyproline in the livers (354.14 ± 141.90 μg/mg) vs. controls (547.80 ± 78.37 μg/mg, *P* < 0.05) (Fig. 5e). Moreover, the levels of pro-fibrotic markers (α-SMA and Col1a1) were significantly lower in the fibrotic livers from the mice treated with AAV8-TBG-FOXA2 than the livers from their control counterparts (Fig. 5f and g). In addition, compared to controls, overexpression FOXA2 in hepatocytes also increased liver weight (Supplementary Fig. 1c). These findings indicated that upregulation of FOXA2 in hepatocytes significantly relieves hepatic fibrosis.

### Pre-overexpression of FOXA2 in HSCs plays an insignificant role in liver fibrosis

**Figure 6:**
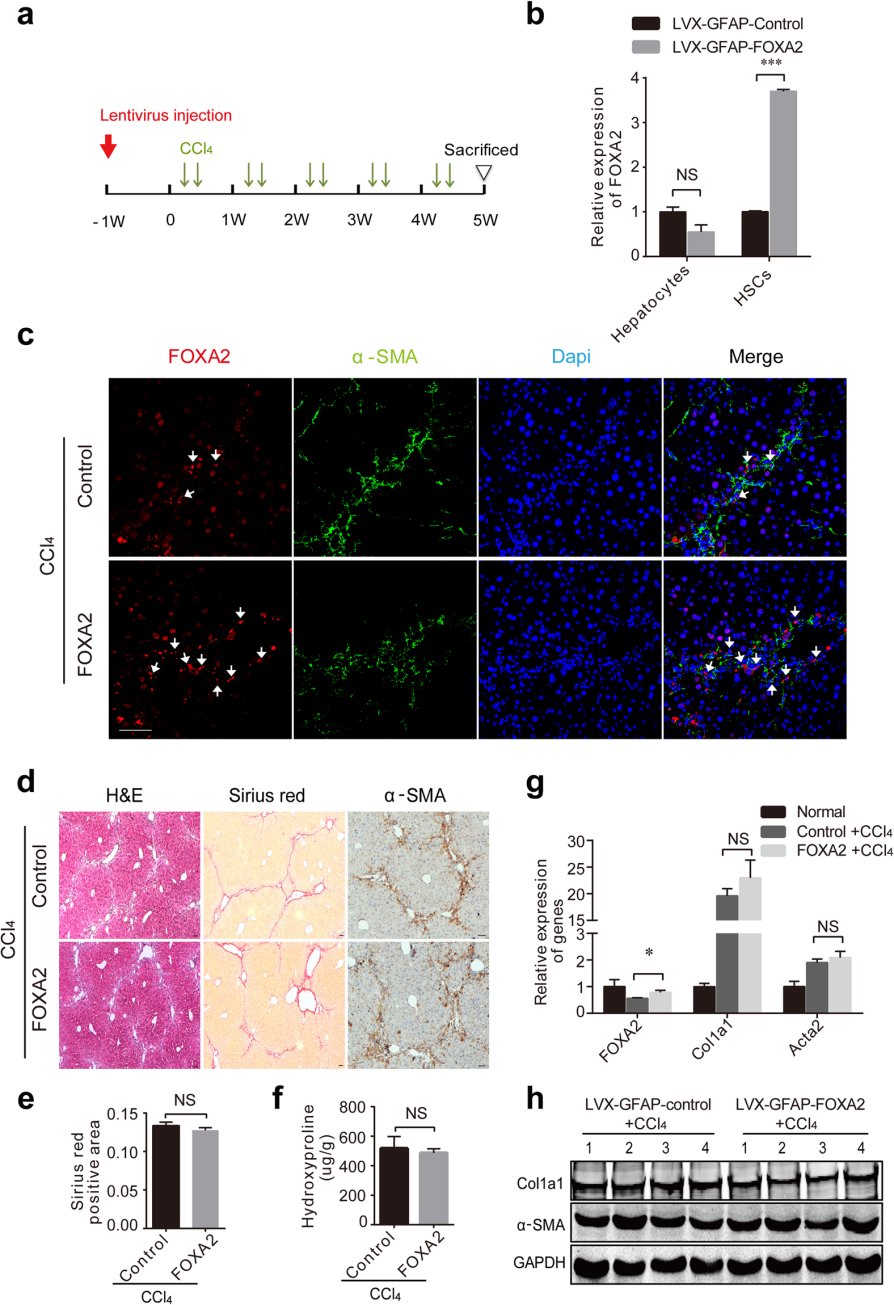
Overexpression of FOXA2 in HSC does not significantly affect liver fibrosis. (**a**) A single dose of LVX-GFAP-FOXA2 was injected via the tail vein one week before CCl_4_ administration. (**b**) FOXA2 mRNA levels in primary hepatocytes and HSCs from LV-GFAP-Control and LV-GFAP-FOXA2 mice were determined by RT-PCR analysis. (**c**) Immunofluorescence showed overexpression of FOXA2 in HSCs in the fibrotic liver (white arrow). (**d**) H&E and Sirius red staining of the fibrotic livers and immunohistochemistry staining of α-SMA. Scale bars, 50 μm. (**e**) Semi-quantitative analysis of Sirius red staining was used to analyse ECM deposition. (**f**) Liver lysates were analysed for hydroxyproline. (**g**) FOXA2, Acta2 and Col1a1 expression in liver tissues from LV-GFAP-Control and LV-GFAP-FOXA2-treated fibrotic mice, as determined by real-time PCR (n = 6 mice in each group). (**h**) The protein levels of Col1a1 and α-SMA in the liver tissues. *P < 0.05, ***P < 0.001.

It is well known that HSCs are the primary effector cells that orchestrate the deposition of ECM in fibrotic livers^1,24^. To explore the role of FOXA2 in HSCs of fibrotic livers, the lentivirus LVX-GFAP-FOXA2 was used to specifically deliver FOXA2 expression under control of the glial fibrillary acidic protein (GFAP) promoter in HSCs. A single dose of LVX-GFAP-FOXA2 or the control virus LVX-GFAP was injected into the mice before CCl_4_ treatment (Fig. 6a). Real-time PCR analysis indicated that FOXA2 expression was enhanced by approximately 3.7 times in HSCs isolated from mice treated with LVX-GFAP-FOXA2 compared with the control mice. No alteration in primary hepatocytes from the two groups was observed (Fig. 6b). After a 5-week CCl_4_ treatment, double immunofluorescence staining also showed intensive expression of FOXA2 located in the activated HSCs of the mice injected with LVX-GFAP-FOXA2 (Fig. 6c). However, both immunofluorescence and immunohistochemical staining showed that LVX-GFAP-FOXA2 did not alter staining of α-SMA in fibrous areas (Fig. 6c and d). Similarly, comparisons between the control group and GFAP-FOXA2 group did not indicate notable differences in ECM deposition (Fig. 6d and e) and hydroxyproline in the livers (516.79 ± 73.94 μg/mg vs 486.66 ± 25.77 μg/mg, *P* > 0.05) (Fig. 6f). Additionally, there was no obvious difference in the expression of Col1a1 and α-SMA between the two groups (Fig. 6g and h). These data demonstrated that FOXA2 upregulation in HSCs has no obvious effect on the pathogenic development of liver fibrosis.

### FOXA2 modulates ER stress in liver fibrosis

**Figure 7:**
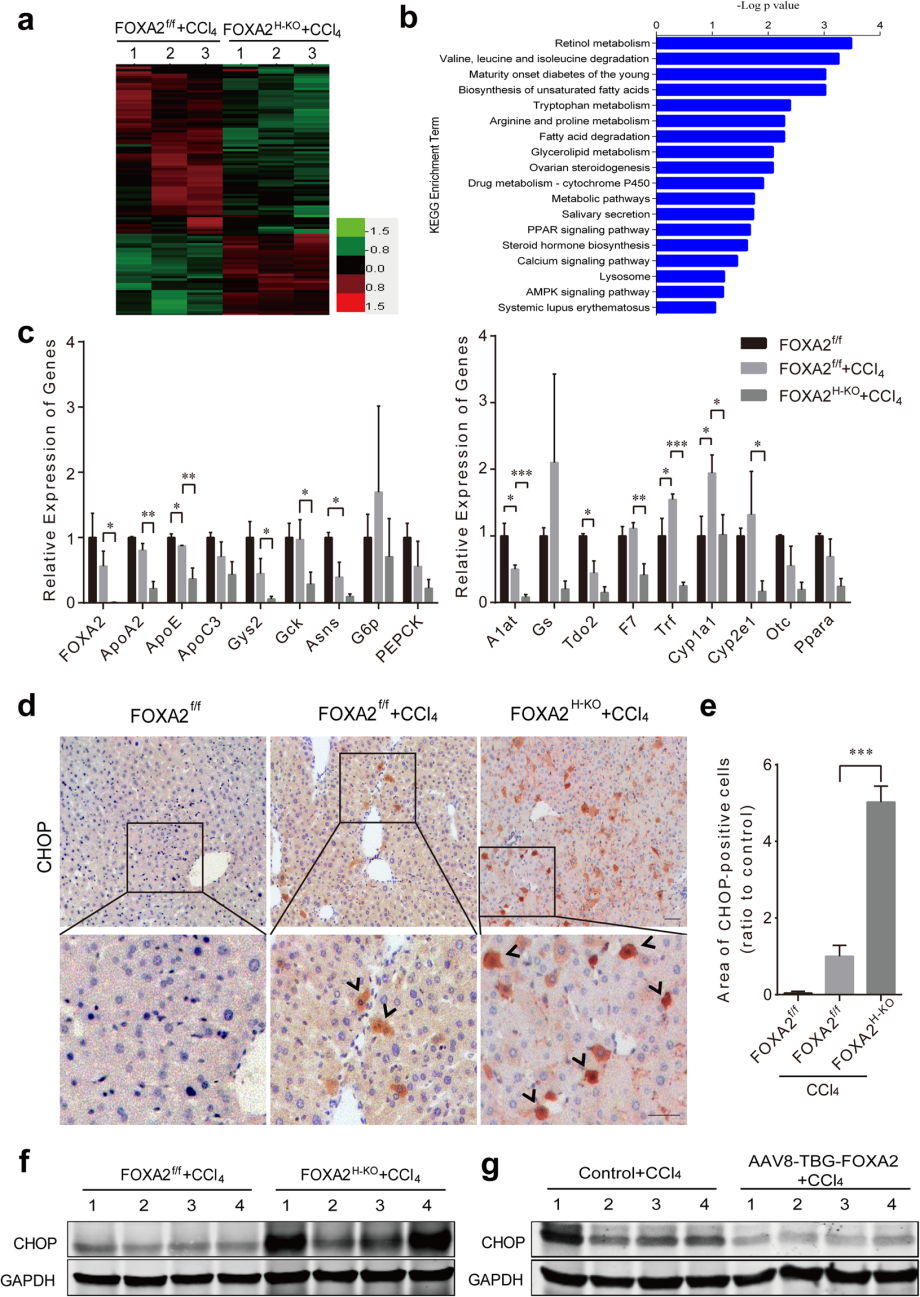
FOXA2 modulates ER stress in hepatocytes during hepatic fibrogenesis. (**a**) A heat map diagram of 544 differentially expressed genes in hepatocytes between FOXA2^f/f^ and FOXA2^H-KO^ fibrotic mice, which were identified by high-throughput RNA-sequence analysis. (**b**) Histogram of KEGG classifications of assembled differential genes. (**c**) Related mRNA levels of the hepatic functional genes in the primary hepatocytes isolated from FOXA2^f/f^ and FOXA2^H-KO^ mice treated with CCl_4_. Left: glucose and lipid metabolism, right: hepatic enzyme synthesis. (n = 4 mice in per biological group). *P < 0.05, **P < 0.01, ***P < 0.001. Apoa2, Apoe and Apoc3, apolipoproteins A2, E and C3; Gys2, glycogen synthase 2; Gck, glucokinase; Asns, asparagine synthetase; G6pc, glucose-6-phosphatase(catalytic); PEPCK, phosphoenolpyruvate carboxykinase; A1at, α1-antitrypsin; Gs, glutamate-ammonia ligase; Tdo2, tryptophan 2,3-dioxygenase; F7, coagulation factor VII; Trf, transferrin; Cyp1a1/2e1, cytochrome P450 1a1/2e1; Otc, ornithine transcarbamylase; Ppara, peroxisome proliferator activated receptor alpha. (**d**) Immunohistochemistry for the ER stress marker CHOP in the livers of FOXA2^f/f^ mice and CCl_4_-treated FOXA2^f/f^ and FOXA2^H-KO^ mice. Scale bars, 50 μm. (**e**) Quantification of the CHOP-positive cells of each group relative to the control. (**f**) Western blot analysis of CHOP in FOXA2^f/f^ and FOXA2^H-KO^ fibrotic livers (n = 6 mice in each group). (**g**) Representative gel image of CHOP expression in livers from CCl_4_-treated mice injected with AAV-TBG-Control or AAV-TBG-FOXA2 (n = 8–10 mice in each group).

Previous studies have demonstrated that FOXA2 plays a vital part in regulating metabolic genes in the liver^16,25^. We performed a high-throughput RNA-sequence analysis of hepatocyte mRNA from FOXA2^f/f^ and FOXA2^H-KO^ fibrotic mice. Setting the significance of a false discovery rate (FDR) at 5% and a fold-change cut-off at 2, we identified 544 differentially expressed genes between control and FOXA2^H-KO^ fibrotic mice (Fig. 7a). Functional classification and pathway assignment were performed in differentially expressed genes by using the Kyoto Encyclopedia of Genes and Genomes (KEGG). A total of 18 top KEGG pathways associated with metabolism were found in this study (Fig. 7b). We next investigated the expression of liver-specific genes involved in glucose, lipid, amino acid, xenobiotic, and drug metabolism in hepatocyte-specific mutant mice. Interestingly, expression of liver metabolic genes in hepatocytes derived from untreated FOXA2^H-KO^ mice showed no obvious differences relative to metabolic gene expression in FOXA2^f/f^ mice (Supplementary Fig. 2b). However, CCl_4_ administration altered the levels of these genes in hepatocytes of FOXA2^f/f^ mice. Importantly, hepatocytes from FOXA2^H-KO^ mice treated with CCl_4_ displayed severely lower expression of hepatic function genes, including apolipoprotein A2 (Apoa2), apolipoprotein E (Apoe), glycogen synthase 2 (Gys2), glucokinase (Gck), coagulation factor VII (F7), transferrin (Trf), cytochrome P450 1a1 and 2e1 (Cyp1a1 and Cyp2e1) (Fig. 7c).

The corelation between metabolism and ER stress has been characterized in several studies^26,27^. In addition, a previous study has also indicated that liver-specific ablation of FOXA2 results in ER stress^19^. Additionally, ER stress has been recognized as being involved in chronic liver disease^28,29^. Therefore, we examined the expression of a molecular marker (CHOP/Gadd153) of ER stress. The protein levels of CHOP showed no significant changes in the livers between FOXA2^H-KO^ and FOXA2^f/f^ mice (Supplementary Fig. 2c). However, immunohistochemistry showed that CHOP expression was slightly elevated in fibrotic livers and further aggravated in hepatocytes with FOXA2 depletion in the mice after CCl_4_ treatment. Distinctly higher CHOP staining was observed in the lobular areas of FOXA2^H-KO^ fibrotic livers (Fig. 7d). The level of CHOP-positive cells in the liver was 5.02-fold higher in CCl_4_-treated FOXA2^H-KO^ mice compared with their control fibrotic littermates (Fig. 7e). In addition, an immunoblot confirmed higher CHOP protein expressed in FOXA2^H-KO^ fibrotic livers relative to the controls (Fig. 7f and Supplementary Fig. 3a), whereas elevation of FOXA2 specifically in hepatocytes significantly repressed the ER stress effect (Fig. 7g and Supplementary Fig. 4a).

### FOXA2 prevents hepatocyte apoptosis in fibrotic livers

**Figure 8:**
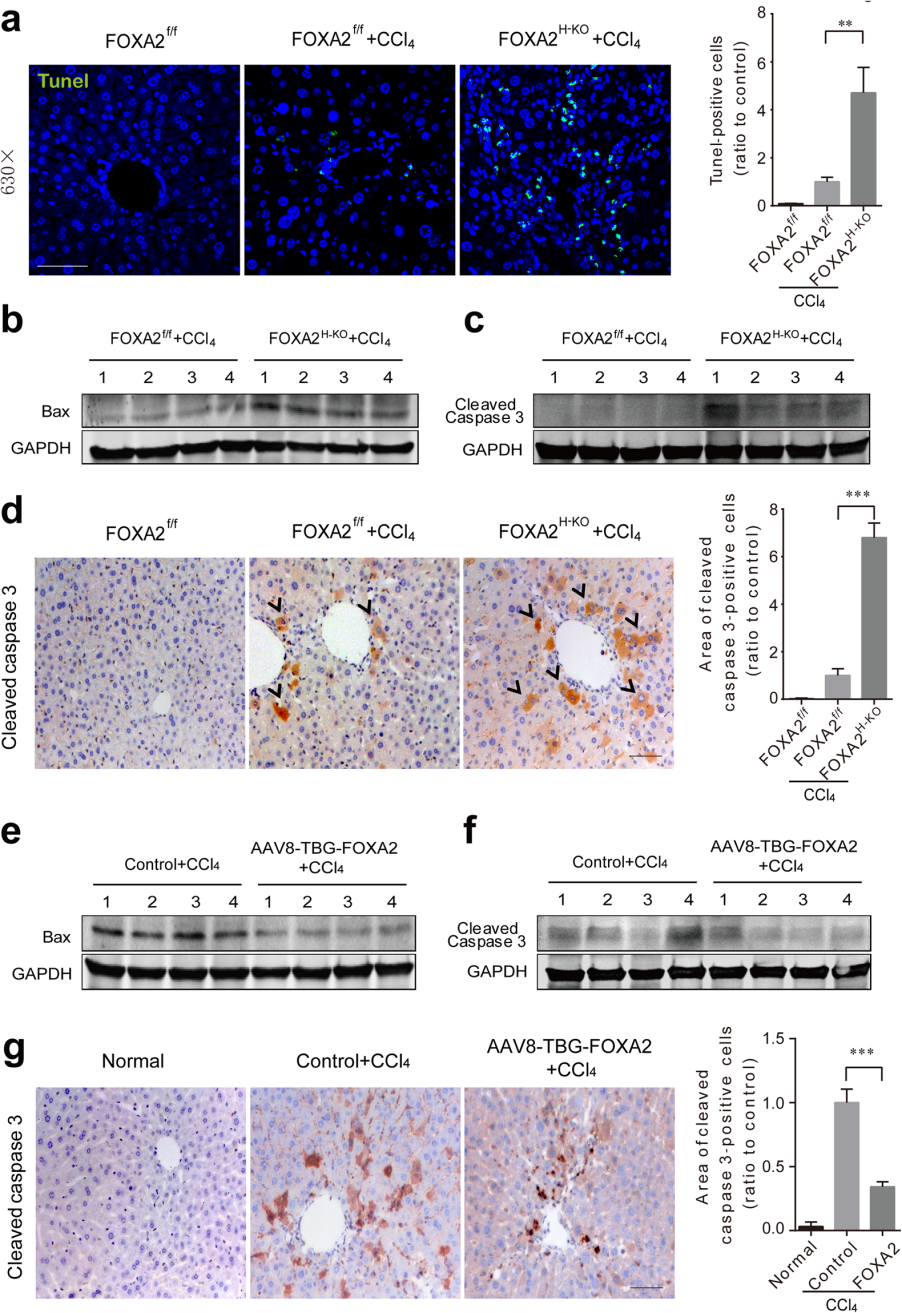
FOXA2 protects hepatocytes against apoptosis in fibrotic livers. (**a**) TUNEL assay indicating the DNA strand breaks in fibrotic livers. The cell nuclei were counterstained using DAPI (blue). Right panel: Quantification of the apoptotic cells. Scale bars, 50 μm. (**b**,**c**) Western blot analysis of the levels of Bax and cleaved caspase-3 in the liver lysates. (**d**) The immunohistochemistry staining for cleaved caspase-3 was quantified by Image-Pro Plus 6 software (right). More cytoplasmic staining for cleaved caspase 3 was observed at the periphery of the centrilobular areas of CCl_4_-treated FOXA2^H-KO^ livers. Representative images are shown (n = 6 mice in per biological group). Scale bars, 50 μm. (**e**,**f**) Western blotting was performed to analyse the levels of apoptosis-regulated proteins, Bax and cleaved caspase 3 from fibrotic liver lysates in AAV-TBG/AAV-TBG-FOXA2 groups. (**g**) The *in situ* analysis of cleaved caspase-3 levels in AAV-TBG-Control or AAV-TBG-FOXA2-treated fibrotic livers (n = 8–10 mice in each group). Data are shown as the mean ± SD. ***P < 0.001.

It is known that prolonged ER stress triggers apoptotic cascades^30,31^. Previous studies have also indicated that FOXA2 is closely associated with hepatic apoptosis^32^. We then measured hepatic apoptosis by using TUNEL assays. More pronounced TUNEL-positive hepatocytes were scattered in the liver lobules of FOXA2^H-KO^ fibrotic mice compared with control fibrotic littermates (Fig. 8a), whereas no spontaneous apoptosis of hepatocytes was detected in FOXA2^H-KO^ mice without CCl_4_ treatment (Supplementary Fig. 2d). To further investigate the signal components of potential apoptotic pathways, the levels of Bax and cleaved caspase 3 were measured. Both were significantly higher in FOXA2^H-KO^ fibrotic livers relative to control livers (Fig. 8b,c and Supplementary Fig. 3b). In addition, the occurrence of apoptosis in FOXA2^H-KO^ fibrotic livers was confirmed by activated caspase 3 staining. Cleaved caspase 3 mostly located in hepatocytes was markedly present at the periphery of the centrilobular areas of CCl_4_-treated FOXA2^H-KO^ mice (Fig. 8d). The data suggested that FOXA2 deletion made hepatocytes more vulnerable to apoptosis during fibrogenesis. Moreover, induction of both Bax and cleaved caspase 3 was attenuated in fibrotic livers with FOXA2 overexpression in hepatocytes (Fig. 8e–g and Supplementary Fig. 4b).

## Discussion

It has been reported that mice lacking FOXA2 die in utero at embryonic days 10–11, owing to severe defects in the development of their cell organization^33^. Bochkis IM *et al*. have demonstrated that Alfp-cre-derived depletion of FOXA2 in both hepatocytes and cholangiocytes of the liver during late gestation disrupts bile acid homeostasis^19^. A recent study by McDaniel K *et al*. has reported that FOXA2 mediates the therapeutic effects of biliary-committed progenitor cells in cholestatic liver disease^20^. All these studies suggest a critical role of FOXA2 in bile duct development. Investigations of the functional role of FOXA2 in hepatocytes have been relatively rare. In this study, we generated a novel mouse model in which FOXA2 was conditionally ablated in mature hepatocytes in adult mice by using AAV8-TBG-Cre. The FOXA2^H-KO^ mice without injury exhibited no obvious alterations in histomorphology (Supplementary Fig. 2a) and the expression levels of the liver-specific metabolic genes. This phenomenon may be explained by the compensatory effect of FOXA1 or other members of HNFs in hepatocytes in which most of the HNFs were highly enriched^34,35^. However, the FOXA2^H-KO^ mice, compared with controls, showed significantly lower expression of hepatic function genes in hepatocytes and more severe fibrosis in livers treated with CCl_4_, thus suggesting that FOXA2 is indispensable for the maintenance of hepatocyte functions in diseased livers and that FOXA2^H-KO^ mice were more predisposed to fibrogenesis.

Hepatocytes, the most abundant cells in the liver, make up 70–85% of the liver mass. The integrity of cellular function of hepatocytes plays a vital role in maintaining the internal homeostasis and regeneration of the liver^36^. In a wide range of hepatic diseases, hepatocytes are the targets of most hepatotoxic agents^37^. Hepatocyte apoptosis is dramatically elevated in patients with non-alcoholic steatohepatitis and alcoholic hepatitis, and the results correlate with hepatic injury and disease severity^38^. Apoptosis of damaged hepatocytes stimulates the fibrogenic actions of liver myofibroblasts^39^. Additionally, ample data indicate that persistent apoptosis of hepatocytes is an essential feature contributing to liver injury and progressive fibrosis^40–42^. In this study, we found that the expression of FOXA2 decreased in hepatocytes but increased in HSCs during liver fibrogenesis. Additionally, conditional deletion of FOXA2 in hepatocytes in adult mice exacerbated the apoptosis of hepatocyte and hepatic fibrogenesis induced by CCl_4_. Furthermore, overexpression of FOXA2 in either the liver or hepatocytes markedly alleviated hepatic apoptosis and liver fibrosis. However, upregulation of FOXA2 in HSCs did not affect the liver fibrogenesis induced by CCl_4_. Therefore, we concluded that FOXA2 inhibits hepatic fibrosis by protecting hepatocytes rather than inhibiting activated HSCs. These data support our previous proposal that restoration of hepatocyte function should be considered a priority for the treatment of liver fibrosis, especially with the upregulation of the HNF family in hepatocytes^10^.

ER stress in hepatocyte injury and chronic liver diseases has received substantial attention in the past few years^29,43^. Aberrant glucose or lipid metabolism can disrupt calcium homeostasis and induce ER stress in the liver^26,27^. A study by Irina M. Bochkis and colleagues has revealed that impaired bile acid homeostasis in mice with liver-specific ablation of FOXA2 at late gestation results in ER stress^19^. In addition, prolonged ER stress may initiate apoptotic cascades and is known to play a predominant role in the pathogenesis of multiple diseases^30,31^. In this study, we confirmed that FOXA2 regulates metabolic genes in hepatocytes during liver injury, in agreement with findings from previous studies^16,25^. Additionally, we showed that loss of FOXA2 in hepatocytes increased CHOP abundance during hepatic fibrogenesis, whereas FOXA2 delivery in hepatocytes reversed the CHOP accumulation in fibrotic livers. On the basis of these findings, we propose that inhibition of ER stress is involved in FOXA2-mediated anti-fibrogenesis effect.

Research has demonstrated that activated HSCs are responsible for fibrogenesis in chronic liver diseases^4^. However, emerging evidence indicates that activated HSCs play an important role in liver development^5^. Although we found that the expression of FOXA2 was enhanced in activated HSCs during fibrogenesis, overexpression of FOXA2 in HSCs did not affect fibrosis. Thus, clarifying the effects of FOXA2 in HSCs in the future would expand understanding of liver diseases. On the other hand, it has been reported that FOXA2 affects bile duct development and biliary-committed progenitor cells in liver regeneration^15,20^. In this study, we also examined whether FOXA2 expression in hepatocytes affects the bile duct reaction during liver fibrogenesis. However, manipulating the expression of FOXA2 in hepatocytes had no significant influence on the bile duct response in fibrotic livers induced by CCl_4_ (Supplementary Fig. 5a and b). Therefore, it is of interest to investigate the effect of FOXA2 alteration in biliary cells on bile duct reaction and fibrosis. In addition, CCl_4_-derived reactive oxygen species (ROS) and free radicals in the liver mainly result in hepatocyte damage^44,45^. Thus, the effects of FOXA2 expression on liver fibrosis induced by biliary injury also needs further investigation.

In summary, the present investigation demonstrated that FOXA2 plays an indispensable role in the maintenance of liver homeostasis in the fibrotic liver. Loss of FOXA2 in hepatocytes renders the liver more vulnerable to fibrogenesis. Overexpression of FOXA2 attenuates liver fibrosis by protecting hepatocytes from ER stress and apoptosis, thus suggesting that restoring hepatocyte integrity by FOXA2 has therapeutic potential for treating chronic liver diseases.

## Materials and Methods

### Human liver samples

Human liver tissues were obtained from the Liver Specimen Repository of the Eastern Hepatobiliary Surgery Hospital of the Second Military Medical University (Shanghai, China). The control liver specimens (*n* = 8) were obtained from healthy livers or patients with hepatic haemangioma. Fibrotic liver specimens (*n* = 30) were obtained from patients with hepatic fibrosis or cirrhosis. The specimens were collected at the time of liver surgery and immediately frozen in liquid nitrogen for real-time PCR. Adult liver sections were fixed in 10% neutral buffered formalin and paraffin embedded. Four-micron sections were obtained for immunohistochemical staining. Informed consent was obtained from all subjects. All experiments on human samples were carried out in strict accordance with the national guidelines and were authorized by the Scientific Investigation Board, Second Military Medical University.

### Viruses

The adeno-associated virus vector pENN-AVV-TBG-PI-RBG containing the hepatocyte-specific thyroid-binding globulin (TBG) promoter was obtained from the Penn Vector Core (p1015-R). The complementary DNA of the Cre gene was cloned into pENN-AVV-TBG-PI-RBG between MluI and SalI sites. A replication-incompetent AAV8-TBG-Cre virus with Cre expression under the control of TBG promoter was packaged and purified by Biowit biotechnologies (Shenzhen, China). In addition, the human FOXA2 gene was inserted between the MluI and SalI sites of pENN-AVV-TBG-PI-RBG to generate the AAV8-TBG-FOXA2 virus for hepatocyte-specific overexpression of FOXA2.

For non-specific FOXA2 upregulation in the liver, the FOXA2 gene was cloned into the lentiviral vector pCDH-CMV-MCS-EF1-copGFP (System Biosciences) to generate the lentivirus LV-CMV-FOXA2. The CMV promoter of pLVX-IRES-ZsGreen1 (PT4064-5, Clontech) was replaced with the hGFAP promoter to establish the HSC-specific expression lentiviral vector pLVX-GFAP-IRES-ZsGreen1. The FOXA2 gene was cloned into the EcoRI and BamHI sites of pLVX-GFAP-IRES-ZsGreen1 to generate lentivirus LVX-GFAP-FOXA2 for specific overexpression of FOXA2 in HSCs. The lentiviral vectors were transfected into subconfluent HEK 293T cells along with the packaging plasmid psPAX2 (Addgene) and envelope plasmid pMD2.G (Addgene) by using FuGENE® 6 transfection reagent (Promega) to produce lentiviral particles. The lentiviruses in the medium were collected 48 h later and concentrated by ultracentrifugation. All vectors were verified by sequencing. The primer sequences are listed in Supplementary Table 2.

### Animals

Eight-week-old male C57Bl/6 J mice (Experimental Center of the Chinese Academy of Sciences, Shanghai, China) were injected intraperitoneally with 0.25 ml/kg CCl_4_ (diluted 1:3 (v/v) in olive oil) biweekly for 4 or 5 weeks to establish the hepatic fibrosis mouse model. FOXA2^flox/flox^ (FOXA2^f/f^) mice were obtained from Jackson Laboratory. A single dose of adeno-associated virus AAV8-TBG-Cre at 2 × 10^11^ genome copies was injected into FOXA2^f/f^ model mice (6 weeks of age) through the tail vein to specifically knockout FOXA2 in hepatocytes (FOXA2^H-KO^). To overexpress FOXA2 in the liver, a single dose of 1 × 10^7^ TU LV-CMV-FOXA2 or control virus was injected into the tail vein 2 weeks after the first CCl_4_ injection. To observe the effects of hepatocyte-specific FOXA2 overexpression on liver fibrosis, the mice were infused with a single dose of 4 × 10^11^ Vg AAV8-TBG-FOXA2 or control virus through the tail vein 1 week after the first CCl_4_ injection. For overexpression of FOXA2 in HSCs, a single dose of 4 × 10^7^ TU lenti-GFAP-FOXA2 or control virus was delivered via the tail vein 1 week prior to the first CCl_4_ injection. The animals were sacrificed on the third day after the last CCl_4_ injection. All animal experiments were carried out in strict accordance with the national guidelines and authorized by the Scientific Investigation Board at Second Military Medical University.

### Isolation of primary hepatocytes and HSCs

Primary mouse hepatocytes were isolated from adult male mice by using a modified version of a two-step collagenase perfusion protocol, as previously described in detail^46^. In brief, mice were anaesthetized, and a V incision was made to expose internal organs. The flushed livers were perfused with DMEM-free plus collagenase IV (1 mg/ml, sigma) following D-Hank’s balanced salt solution including EDTA (0.5 mM). After perfusion, the livers were removed from the mice, and the digested hepatocytes were dispersed in DMEM-free and filtered through 80 and 200 mesh sieves to remove the undigested debris. The filtrates were centrifuged at 300 rpm for 5 minutes at 4 °C. The hepatocytes in the precipitate were washed with DMEM-free 3 times and harvested for subsequent analysis.

The protocol for primary mouse HSC isolation was similar to the methods mentioned above. The flushed livers were perfused with DMEM-free containing collagenase IV (1 mg/ml) and pronase (2 mg/ml, Roche) following D-Hank’s balanced salt solution including EDTA (0.5 mM). After perfusion, the livers were removed, and the digested hepatic cells were dispersed in DMEM-free. Next, DNA enzymes were added to prevent filamentous gelatinous material, and the undigested debris was removed through a filter. The filtrates were centrifuged at 300 rpm in a centrifuge tube for 5 minutes at 4 °C. The supernatant was collected following gradient centrifugation with 25% Nycodenz (Sigma) to isolate primary HSCs. Cells were washed and plated on 60-mm-diameter tissue culture dishes. After 4 h of incubation at 37 °C, the adherent HSCs were harvested for further analysis.

### RNA isolation and quantitative real-time polymerase chain reaction (PCR) analysis

Total RNA was extracted from liver tissues, primary hepatocytes and HSCs with ready-to-use TRIZOL Reagent (TaKaRa). First-strand cDNA was synthesized from 1 *μ*g total RNA using PrimeScript™ RT Master Mix (Takara). cDNA was quantified by qPCR using SYBR Green PCR Kit (TaKaRa) with the specific primers. Expression was normalized to beta actin. Primers used are listed in Supplementary Table 1.

### Histology, immunohistological and immunofluorescence analysis

For histopathological examination, the liver tissue sections were stained with haematoxylin-eosin (H&E). Sirius red was used to stain for collagen. Immunohistochemistry was performed using 4–5 mm paraffin-embedded thick liver sections, and observed under a photomicroscope. Immunofluorescence staining were observed under a laser scanning confocal fluorescence microscope (Carl Zeiss, Jena, Germany). Antibodies were used as follow: FOXA2 (ab108422, Abcam), α-SMA (BM0002, Boster), α-SMA (MO85129, Dako), CHOP (sc-575, Santa Cruz) and Cleaved Caspase 3 (Asp175,9664, Cell Signaling). Areas of positive stained sites were measured by using image analyses software Image-Pro Plus 6.0 (Media Cybernetics).

### Western blot assay

Cells or liver tissues were collected with RIPA buffer (Beyotime) containing a protease inhibitor cocktail (cocktail B14001) and a phosphatase inhibitor cocktail (cocktail B15001). Proteins were run on a 4–15% polyacrylamide gel followed by transferring to a methanol-activated NC membrane (Millipore). The membrane was blocked in PBS-T containing 5% milk for 1 h prior to incubation with a primary antibody overnight at 4 °C. After 2 h incubation with donkey-anti-mouse or goat-anti-rabbit secondary antibody (IRDye 800), signals were detected by using an Odyssey infrared imaging system (LI-COR) at a wavelength of 800 nm. The primary antibodies used included FOXA2 (ab108422, Abcam), collagen I (BAO325, Boster), α-SMA (BM0002, Boster), CHOP (L63F7, 2895, Cell Signaling), Bax (sc-526, Santa Cruz), Cleaved Caspase 3 (Asp175,9664, Cell Signaling) and GAPDH (BSAP0063, Bioworld).

### Measurement of hydroxyproline content

One hundred mg of wet liver tissue for each sample was subjected to acid hydrolysis to determine the amount of hydroxyproline according to the protocol of the Hydroxyproline Testing Kit (A030-2, Jiancheng, Nanjing, China).

### RNA-Sequencing

Total RNA was isolated using an RNeasy mini kit (Qiagen, Germany). Paired-end libraries were synthesized by using a TruSeq® RNA Sample Preparation Kit (Illumina, USA) according to the TruSeq® RNA Sample Preparation Guide. Briefly, poly-A-containing mRNAs were purified using poly-T oligo-attached magnetic beads. After purification, the mRNA was fragmented into small pieces by using divalent cations under 94 °C for 8 min. The cleaved RNA fragments were copied into first strand cDNA using reverse transcriptase and random primers. This procedure was followed by second strand cDNA synthesis using DNA Polymerase I and RNase H. These cDNA fragments were subjected to an end repair process, the addition of a single ‘A’ base, and ligation of the adapters. The products were then purified and enriched with PCR to create the final cDNA library. Purified libraries were quantified with a Qubit® 2.0 Fluorometer (Life Technologies, USA) and validated with an Agilent 2100 bioanalyzer (Agilent Technologies, USA) to confirm the insert size and calculate the molar concentration. The cluster was generated with a cBot with the library diluted to 10 Pm, and then the samples were sequenced on an Illumina HiSeq System (Illumina, USA). The library construction and sequencing was performed at Shanghai Biotechnology Corporation. The entire dataset is available at NCBI Gene Expression Omnibus (http://www.ncbi.nlm.nih.gov/geo/) under accession number GSE97344.

### TUNEL assay

TUNEL (terminal deoxynucleotidyl transferase dUTP nick-end labelling) staining was performed using a TdT-FragEL™ DNA fragmentation detection kit (C1086, Beyotime) or an *In Situ* Cell Death Detection Kit (Roche Diagnostics, Indianapolis, USA) according to the manufacturers’ protocols. The nuclei were labelled with 4′,6-diamidino-2-phenylindole (DAPI) and then visualized under a confocal microscope.

### Statistical analysis

The results are presented as the mean ± SD. Two-sided independent Student’s t tests were performed to analyse gene or protein expression levels, hydroxyproline content and histology data. Data on location parameters (median) were analysed using Mann-Whitney tests.

### Data availability statement

All data generated or analyzed during this study are included in this published article and its Supplementary Information files.

## Electronic supplementary material

Supplementary information

## References

[1] Lee UE, Friedman SL (2011). Mechanisms of hepatic fibrogenesis. Best Pract Res Clin Gastroenterol.

[2] Koyama Y, Brenner DA (2017). Liver inflammation and fibrosis. J Clin Invest.

[3] Bataller R, Brenner DA (2005). Liver fibrosis. J Clin Invest.

[4] Hellerbrand C (2013). Hepatic stellate cells–the pericytes in the liver. Pflugers Arch.

[5] Yin C, Evason KJ, Asahina K, Stainier DY (2013). Hepatic stellate cells in liver development, regeneration, and cancer. J Clin Invest.

[6] Suzuki A, Iwama A, Miyashita H, Nakauchi H, Taniguchi H (2003). Role for growth factors and extracellular matrix in controlling differentiation of prospectively isolated hepatic stem cells. Development.

[7] Bansal MB (2016). Hepatic stellate cells: fibrogenic, regenerative or both? Heterogeneity and context are key. Hepatol Int.

[8] Kordes C, Sawitza I, Götze S, Herebian D, Häussinger D (2014). Hepatic stellate cells contribute to progenitor cells and liver regeneration. J Clin Invest.

[9] Schrem H, Klempnauer J, Borlak J (2002). Liver-enriched transcription factors in liver function and development. Part I: the hepatocyte nuclear factor network and liver-specific gene expression. Pharmacol Rev.

[10] Qian H (2015). An HNF1α-regulated feedback circuit modulates hepatic fibrogenesis via the crosstalk between hepatocytes and hepatic stellate cells. Cell Res.

[11] Yue HY (2010). Hepatocyte nuclear factor 4alpha attenuates hepatic fibrosis in rats. Gut.

[12] Nishikawa T (2015). Resetting the transcription factor network reverses terminal chronic hepatic failure. J Clin Invest.

[13] Friedman JR, Kaestner KH (2006). The Foxa family of transcription factors in development and metabolism. Cell Mol Life Sci.

[14] Lee CS, Friedman JR, Fulmer JT, Kaestner KH (2005). The initiation of liver development is dependent on Foxa transcription factors. Nature.

[15] Li Z (2009). Foxa1 and Foxa2 regulate bile duct development in mice. J Clin Invest.

[16] Wolfrum C, Asilmaz E, Luca E, Friedman JM, Stoffel M (2004). Foxa2 regulates lipid metabolism and ketogenesis in the liver during fasting and in diabetes. Nature.

[17] Nakamura K, Moore R, Negishi M, Sueyoshi T (2007). Nuclear pregnane X receptor cross-talk with FoxA2 to mediate drug-induced regulation of lipid metabolism in fasting mouse liver. J Biol Chem.

[18] Wang K (2015). Molecular mechanisms of hepatic apoptosis regulated by nuclear factors. Cell Signal.

[19] Bochkis IM (2008). Hepatocyte-specific ablation of Foxa2 alters bile acid homeostasis and results in endoplasmic reticulum stress. Nat Med.

[20] McDaniel K (2017). Forkhead box A2 regulates biliary heterogeneity and senescence during cholestatic liver injury in mice‡. Hepatology.

[21] Li Z, Tuteja G, Schug J, Kaestner KH (2012). Foxa1 and Foxa2 are Essential for Sexual Dimorphism in Liver Cancer. Cell.

[22] Wang J (2014). FOXA2 suppresses the metastasis of hepatocellular carcinoma partially through matrix metalloproteinase-9 inhibition. Carcinogenesis.

[23] Mu X (2015). Hepatocellular carcinoma originates from hepatocytes and not from the progenitor/biliary compartment. J Clin Invest.

[24] Wynn TA (2008). Cellular and molecular mechanisms of fibrosis. J Pathol.

[25] Rausa FM (2000). Elevated levels of hepatocyte nuclear factor 3beta in mouse hepatocytes influence expression of genes involved in bile acid and glucose homeostasis. Mol Cell Biol.

[26] Zheng, J. *et al*. Docosahexaenoic Acid Ameliorates Fructose-Induced Hepatic Steatosis Involving ER Stress Response in Primary Mouse Hepatocytes. *Nutrients* **8** (2016). 10.3390/nu8010055 PMC4728666 26805874

[27] Fu S (2011). Aberrant lipid metabolism disrupts calcium homeostasis causing liver endoplasmic reticulum stress in obesity. Nature.

[28] Ozcan U (2006). Chemical chaperones reduce ER stress and restore glucose homeostasis in a mouse model of type 2 diabetes. Science.

[29] Tamaki N (2008). CHOP deficiency attenuates cholestasis-induced liver fibrosis by reduction of hepatocyte injury. Am J Physiol Gastrointest Liver Physiol.

[30] Malhotra JD, Kaufman RJ (2007). Endoplasmic reticulum stress and oxidative stress: a vicious cycle or a double-edged sword. Antioxid Redox Signal.

[31] Uzi D (2013). CHOP is a critical regulator of acetaminophen-induced hepatotoxicity. J Hepatol.

[32] Wang K, Brems JJ, Gamelli RL, Holterman AX (2013). Foxa2 may modulate hepatic apoptosis through the cIAP1 pathway. Cell Signal.

[33] Weinstein DC (1994). The winged-helix transcription factor HNF-3 beta is required for notochord development in the mouse embryo. Cell.

[34] Sund NJ (2000). Hepatocyte nuclear factor 3beta (Foxa2) is dispensable for maintaining the differentiated state of the adult hepatocyte. Mol Cell Biol.

[35] Duncan SA, Navas MA, Dufort D, Rossant J, Stoffel M (1998). Regulation of a transcription factor network required for differentiation and metabolism. Science.

[36] Malato Y (2011). Fate tracing of mature hepatocytes in mouse liver homeostasis and regeneration. J Clin Invest.

[37] Higuchi H, Gores GJ (2003). Mechanisms of liver injury: an overview. Curr Mol Med.

[38] Ribeiro PS (2004). Hepatocyte apoptosis, expression of death receptors, and activation of NF-kappaB in the liver of nonalcoholic and alcoholic steatohepatitis patients. Am J Gastroenterol.

[39] Canbay A, Friedman S, Gores GJ (2004). Apoptosis: the nexus of liver injury and fibrosis. Hepatology.

[40] Inokuchi S (2010). Disruption of TAK1 in hepatocytes causes hepatic injury, inflammation, fibrosis, and carcinogenesis. Proc Natl Acad Sci USA.

[41] Takehara T (2004). Hepatocyte-specific disruption of Bcl-xL leads to continuous hepatocyte apoptosis and liver fibrotic responses. Gastroenterology.

[42] Vick B (2009). Knockout of myeloid cell leukemia-1 induces liver damage and increases apoptosis susceptibility of murine hepatocytes. Hepatology.

[43] Guo HL (2017). Pyrazinamide-induced hepatotoxicity is alleviated by 4-PBA via inhibition of the PERK-eIF2α-ATF4-CHOP pathway. Toxicology.

[44] Lushchak VI (2014). Free radicals, reactive oxygen species, oxidative stress and its classification. Chem Biol Interact.

[45] Chen S, Zou L, Li L, Wu T (2013). The protective effect of glycyrrhetinic acid on carbon tetrachloride-induced chronic liver fibrosis in mice via upregulation of Nrf2. PLoS One.

[46] Williams GM, Laspia MF, Dunkel VC (1982). Reliability of the hepatocyte primary culture/DNA repair test in testing of coded carcinogens and noncarcinogens. Mutat Res.

