# Supplementary material for: FOXA2 alleviates CCl4-induced liver fibrosis by protecting hepatocytes in mice
Source: Sci Rep. 2017 Nov 14;7:15532. doi: 10.1038/s41598-017-15831-6 (PMC5686201; doi:10.1038/s41598-017-15831-6)
Supplement: Supplementary file 1 — Supplementary information [file 41598_2017_15831_MOESM1_ESM.pdf]

FOXA2 alleviates CCl_4-induced liver fibrosis by protecting hepatocytes in mice

Wei Wang, Li-Jia Yao, Weifeng Shen, Kai Ding, Pei-Mei Shi, Fei Chen, Jin He, Jin Ding, Xin Zhang, Wei-Fen Xie

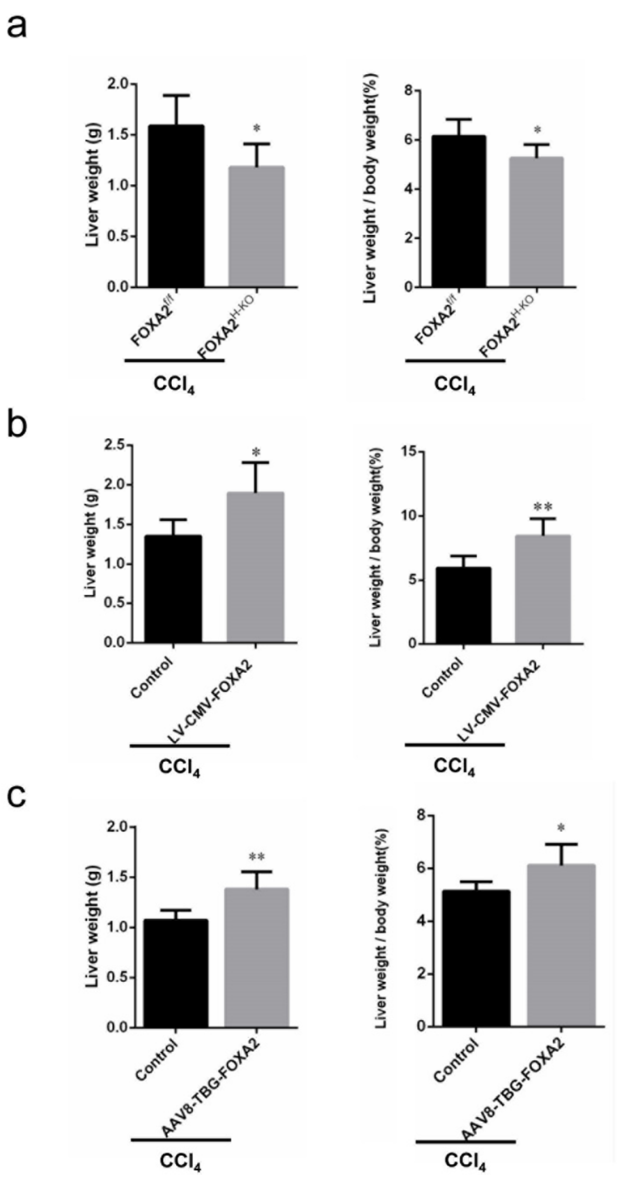

Supplementary Figure 1. FOXA2 affects the liver weight and the liver/body weight ratios in the fibrotic mice.

(a) Knockout FOXA2 in hepatocytes decreased the liver weight and the liver/body weight ratios in the mice treated with CCl_4 (n=6 mice in each group). (b) Overexpression of FOXA2 in livers increased the liver weight and the liver/body weight ratios in the fibrotic mice (n=6 mice in each group). (c) Overexpression of FOXA2 in hepatocytes increased the liver weight and the liver/body weight ratios in the fibrotic mice. (n = 8-10 mice in each group). *$P<0.05$, **$P<0.01$.

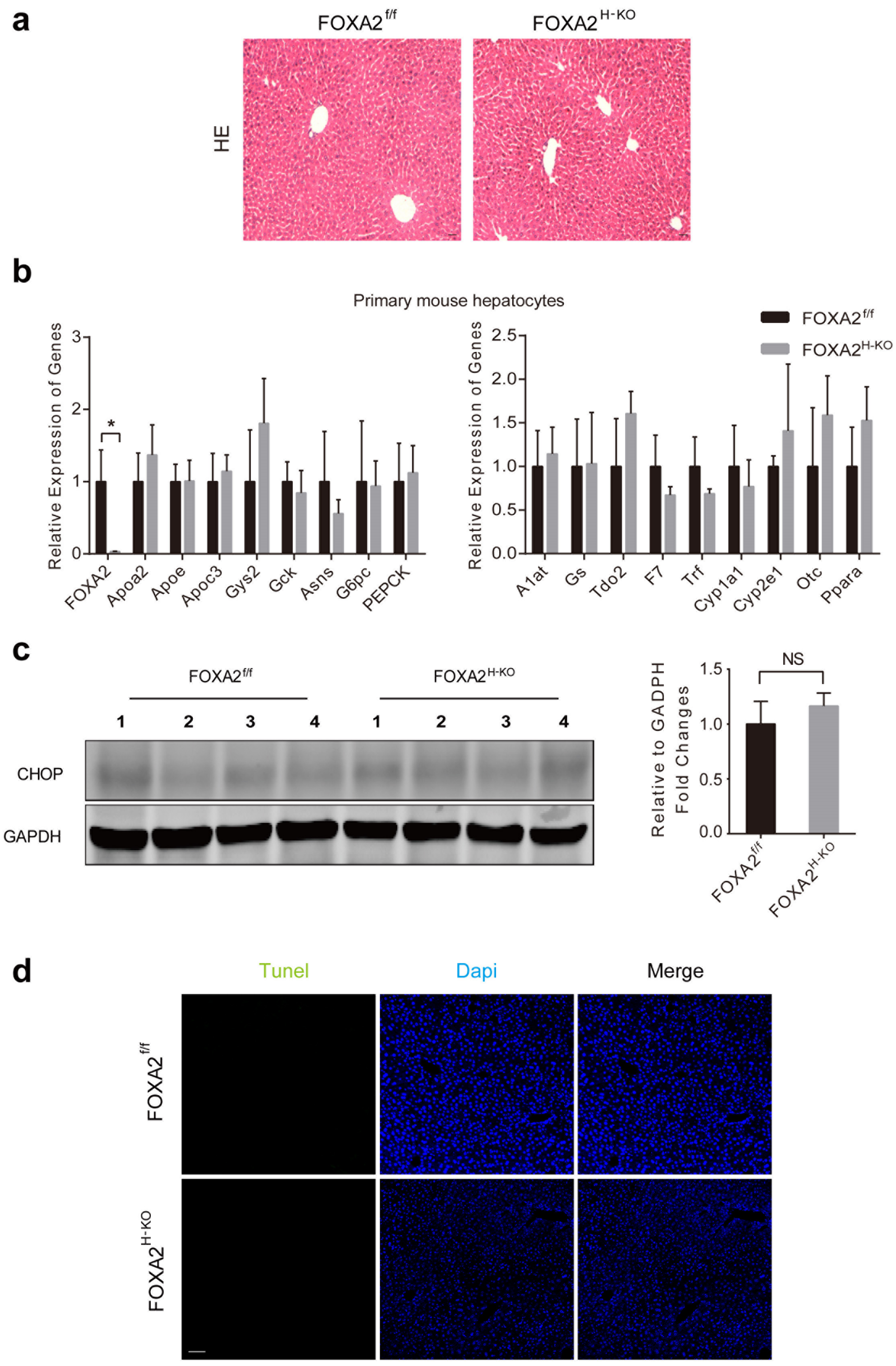

Supplementary Figure 2. FOXA2^{H-KO} mice without CCl_4 treatment show no spontaneous hepatic injury and apoptosis.

(a) Routine H&E stain was carried out to analysis the liver histology and morphology of untreated FOXA2$^{f/f}$ and FOXA2^{H-KO} animals. No necrosis and inflammatory cell infiltration

has been observed in the livers of FOXA2$^{f/f}$ and FOXA2$^{H\text{-}KO}$ mice. Scale bars, 50 μm. (b) Expression of hepatic functional genes in primary hepatocytes from FOXA2$^{f/f}$ and FOXA2$^{H\text{-}KO}$ mice (n=4). (c) The protein level of CHOP in liver lysate was measured by Western blotting (n=4). (d) TUNEL assay shows that no apoptosis occurred in the liver of FOXA2$^{f/f}$ and FOXA2$^{H\text{-}KO}$ mice without CCl_4 treatment. Scale bars, 50 μm. *P<0.05.

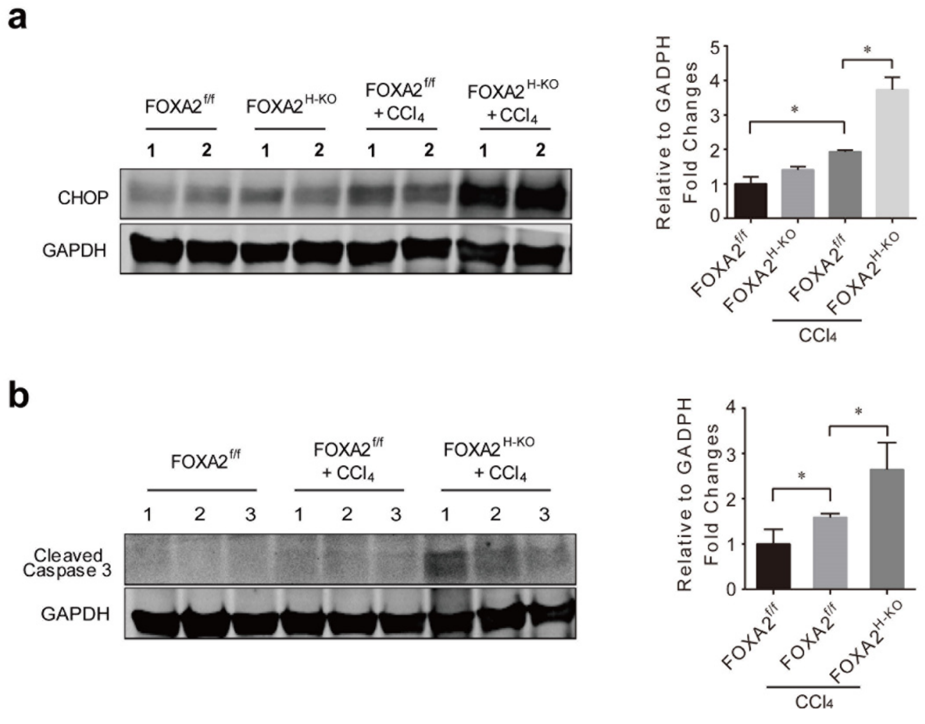

Supplementary Figure 3 FOXA2 deficiency in hepatocytes enhances ER stress and cell apoptosis in fibrotic livers.

(a) Western blotting for ER stress sign (CHOP) in FOXA2$^{f/f}$ and FOXA2$^{H\text{-}KO}$ mouse livers with or without CCl_4 injection as well as its quantification. (b) Representative gel image of Western blotting for the cleaved caspase 3 in livers. The ratio of cleaved caspase 3 was evaluated (n=6). *$P < 0.05$.

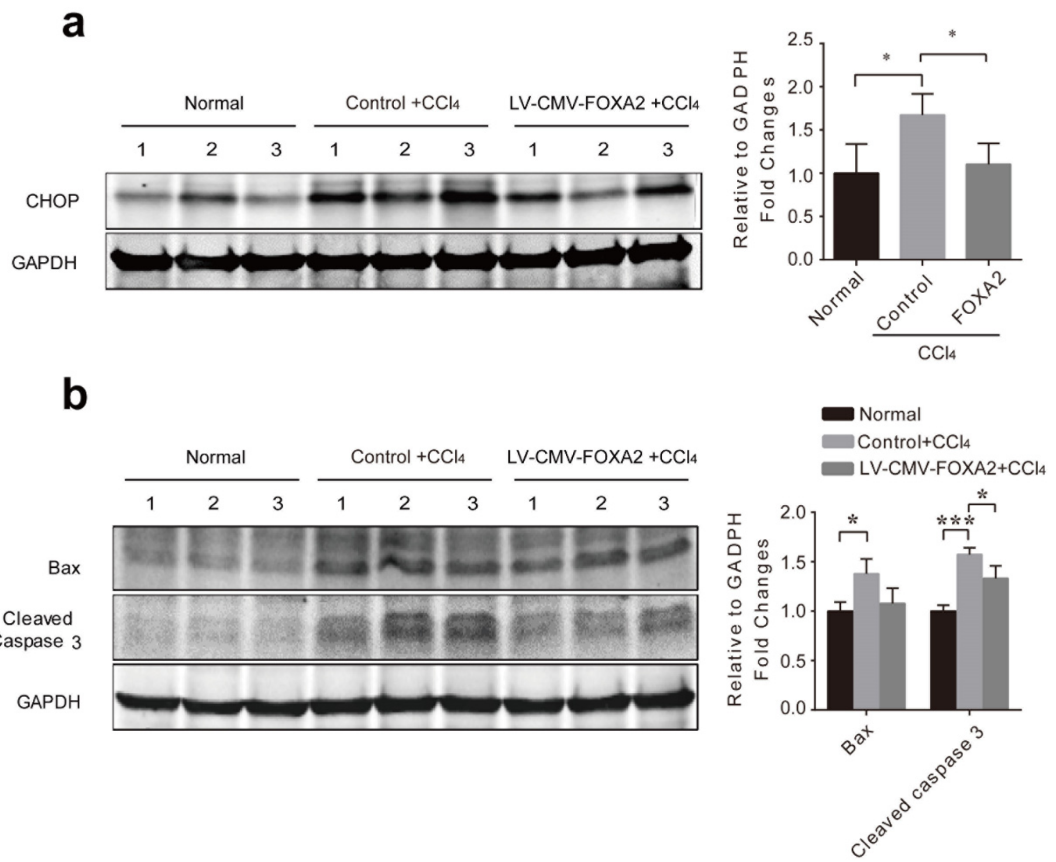

Supplementary Figure 4. Upregulation of FOXA2 in liver inhibits ER stress and hepatic apoptosis during liver fibrogenesis.

(a, b) Western blot was performed to analyze (a) CHOP and (b) apoptosis-related proteins (Bax and cleaved caspase 3) from liver lysates in LV-CMV-Control and LV-CMV-FOXA2-treated mice with CCl_4 administration and then calculated with software Image J (n=6). **P*<0.05, ****P* < 0.001.

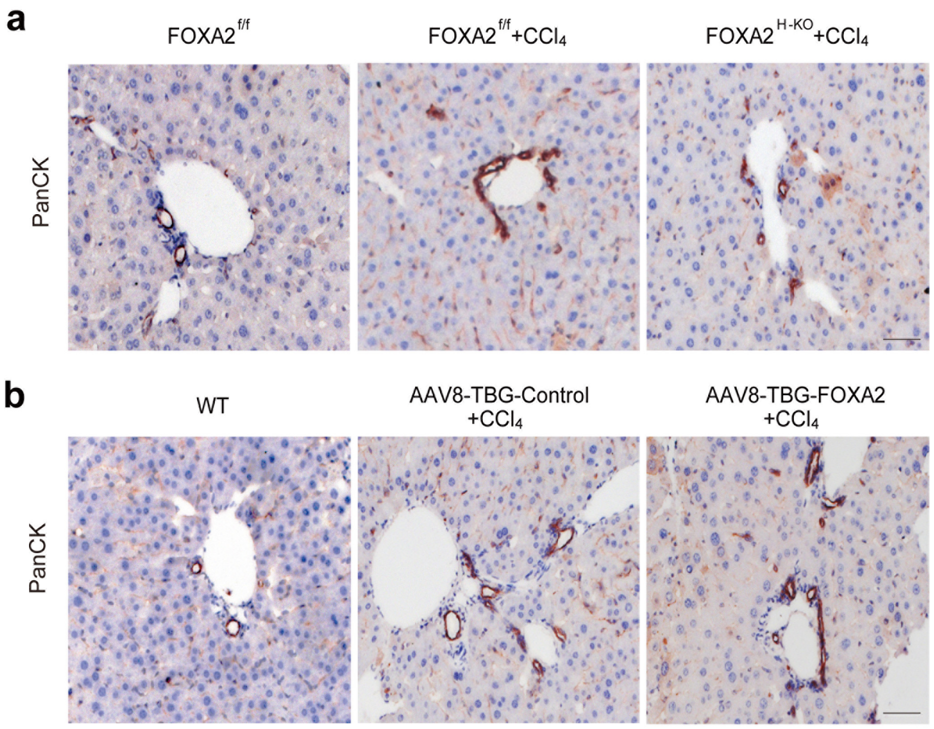

Supplementary Figure 5. The expression of FOXA2 in hepatocytes has no effect on the bile duct response in fibrotic livers induced by CCl_4.

(a, b) Immunohistochemistry staining of PanCK was applied to determine the duct reaction of the fibrotic livers from $FOXA2^{f/f}$ and $FOXA2^{H\text{-}KO}$ mice (a) or AAV-TBG or AAV8-TBG-FOXA2-treated mice (b). Scale bars, 50 μm.

Supplementary Table 1. Primers used for Real-time PCR

Gene	Forward primer (5'- 3')	Reverse primer (5'- 3')
Human FOXA2	TTCTTTCCCGTTTTCCTCCTTAT	GAGAAGAAATCCATAACACCCCC
Mouse FOXA2	AGCACCATTACGCCTTCAAC	CCTTGAGGTCCATTTTGTGG
Mouse Col1α1	TAAAGGGTCATCGTGGCTTC	GACGGCTGAGTAGGGAACAC
Mouse Acta2	CTGTCCCTCTATGCCTCTGG	AGGGCTGTGATCTCCTTCTG
Mouse Apoa2	CCATCTGTAGCCTGGAAGGA	TTGGCCTTCTCCATCAAATC
Mouse Apoe	AACCGCTTCTGGGATTACCT	TTCCGTCATAGTGTCCTCCA
Mouse Apoc3	GGGTACTCCTTGTTGTTGC	AAATCCCAGAACTCAGAGAAC
Mouse Gys2	CATGCCAGACACCTGACACT	CGGAGAAGGTGGTACTGAGG
Mouse Gck	CCAGAAGGCTCAGAAGTTGG	TCTGGTGTTTCGTCTTCACG
Mouse Asns	ATGACAAAGGAGGCATCGAG	AAGGGCCTGACTCCATAGGT
Mouse G6pc	GGCTCCATGACTGTGGGATC	TTCAGCTGCACAGCCCAGAA
Mouse PEPCK	GTGTCCCTCTAGTCTATGAAGC	ATTGACTTGATCCTCCAGATAC
Mouse A1at	AGGCTGACATCCACAAGTCC	CTCCACCAGCTTCAGGTCAT
Mouse Gs	CCTGCTTGTATGCTGGAGTC	GAAAAGTCGTTGATGTTGGA
Mouse Tdo2	TGAAAGGCCTGGAAGAAGAA	CGCTTCTCATCAAACAAGCA
Mouse F7	ACGCCAGATGGATAGTGACC	ACCCGTCGTACTTGCTCATC
Mouse Trf	CCGGGTTAAGGCTGTACTGA	TTTGGTGTCATCCCTGAACA
Mouse Cyp1a1	CCTGGCTGTCACCGTATTCT	CAGCATGTGACCAATGAAGG
Mouse Cyp2e1	AGGCTGTCAAGGAGGTGCTA	CCTTCCATGTGGGTCCATTA
Mouse Otc	CTGCAGAAGGAGCTAGAACAGT	AGAACCATACTCGAACCACATC
Mouse Ppara	AATGCAATTCGCTTTGGAAG	TTGCCCAGAGATTTGAGGTC
β-actin	TGTCACCAACTGGGACGATA	GGTCTTTACGGATGTCAACG

Supplementary Table 2. Primers used for the construction of recombinant plasmid

Gene Symbol	Cloning Vector	Forward primer (5'- 3')	Reverse primer (5'- 3')
Cre	pENN.AAV.TBG.PI.RBG	CGGACGCGTGCCACCATGTCCAATTTACTGACCGTACACCAAAAT	CGCGTCGACCTAATCGCCATCTTCCAGCAGGCGCACCA
hFOXA2	pENN.AAV.TBG.PI.RBG	CGGACGCGTGCCACCATGCACTCGGCTTCCAGTATGCT	CGCGTCGACTTAAGAGGAGTTCATAATGGGCCGGGAGT
hFOXA2	pCDH-CMV-MCS-EF1-copGFP	CCGGAATTCATGCACTCGGCTTCCAGTA	CGCGGATCCTTAAGAGGAGTTCATAATGGGC
hGFAP	pLVX-IRES-ZsGreen1	CCCATCGATGAGCTCCCACCTCCCTCTCTGTGCTGGGACTCAC	CCGGAATTCCCTGCTCTGGCTCTGCTCGCTCCTGGGATG
hFOXA2	pLVX-hGFAP-IRES-ZsGreen1	CCGGAATTCGCCACCATGCACTCGGCTTCCAGTATGCT	CGCGGATCCTTAAGAGGAGTTCATAATGGGCCGGGAGT
